# Contribution of thirdhand smoke to overall tobacco smoke exposure in pediatric patients: study protocol

**DOI:** 10.1186/s12889-019-6829-7

**Published:** 2019-05-02

**Authors:** E. Melinda Mahabee-Gittens, Georg E. Matt, Eunha Hoh, Penelope J. E. Quintana, Lara Stone, Maegan A. Geraci, Chase A. Wullenweber, Gena N. Koutsounadis, Abigail G. Ruwe, Gabriel T. Meyers, Mark A. Zakrajsek, John K. Witry, Ashley L. Merianos

**Affiliations:** 1 Divison of Emergency Medicine, Cincinnati Children’s Hospital Medical Center, 3333 Burnet Avenue MLC 2008, Cincinnati, Ohio 45229-3039 USA; 2 University of Cincinnati College of Medicine, CARE/Crawley Building, Suite E-870 3230 Eden Avenue, Cincinnati, Ohio 45267 USA; 3 Department of Psychology, San Diego State University, San Diego, CA USA; 4 San Diego State University Graduate School of Public Health, San Diego, CA USA; 5 School of Human Services, University of Cincinnati, PO Box 210002, Cincinnati, Ohio 45221 USA

**Keywords:** Tobacco, Secondhand smoke, Thirdhand smoke, Pediatrics, Emergency department, Urgent care, Smoking cessation

## Abstract

**Background:**

Thirdhand smoke (THS) is the persistent residue resulting from secondhand smoke (SHS) that accumulates in dust, objects, and on surfaces in homes where tobacco has been used, and is reemitted into air. Very little is known about the extent to which THS contributes to children’s overall tobacco smoke exposure (OTS) levels, defined as their combined THS and SHS exposure. Even less is known about the effect of OTS and THS on children’s health. This project will examine how different home smoking behaviors contribute to THS and OTS and if levels of THS are associated with respiratory illnesses in nonsmoking children.

**Methods:**

This project leverages the experimental design from an ongoing pediatric emergency department-based tobacco cessation trial of caregivers who smoke and their children (NIHR01HD083354). At baseline and follow-up, we will collect urine and handwipe samples from children and samples of dust and air from the homes of smokers who smoke indoors, have smoking bans or who have quit smoking. These samples will be analyzed to examine to what extent THS pollution at home contributes to OTS exposure over and above SHS and to what extent THS continues to persist and contribute to OTS in homes of smokers who have quit or have smoking bans. Targeted and nontargeted chemical analyses of home dust samples will explore which types of THS pollutants are present in homes. Electronic medical record review will examine if THS and OTS levels are associated with child respiratory illness. Additionally, a repository of child and environmental samples will be created.

**Discussion:**

The results of this study will be crucial to help close gaps in our understanding of the types, quantity, and clinical effects of OTS, THS exposure, and THS pollutants in a unique sample of tobacco smoke-exposed ill children and their homes. The potential impact of these findings is substantial, as currently the level of risk in OTS attributable to THS is unknown. This research has the potential to change how we protect children from OTS, by recognizing that SHS and THS exposure needs to be addressed separately and jointly as sources of pollution and exposure.

**Trial registration:**

ClinicalTrials.gov Identifier: NCT02531594. Date of registration: August 24, 2015.

## Background

Thirdhand smoke (THS) is the persistent residue resulting from secondhand smoke (SHS) that accumulates in dust, objects, and on surfaces in homes where tobacco has been used, and is re-emitted into the air [1, 2]. Our research indicates that smoker’s homes become reservoirs of persistent toxic pollutants, such as nicotine, polycyclic aromatic hydrocarbons (PAH), and the highly carcinogenic tobacco-specific nitrosamines (TSNAs). Some THS pollutants continue to undergo further chemical changes in the home. Specifically, semi-volatile tobacco smoke compounds can adsorb into carpets, furnishings, and walls where they may react with ambient oxidants (e.g., nitrous acid) to create novel toxicants, some of which are human carcinogens [1–4]. Children are exposed to THS toxicants via inhalation, ingestion, and dermal transfer from THS reservoirs, and children of smokers carry THS pollutants on their hands [5–7]. Children are more sensitive to pollutants than adults [8–10] and much more exposed to house dust than adults [11–13]. Even in homes with smoking bans, children have 5-7 times more nicotine exposure than in nonsmoking homes [5]; levels are higher in apartments [14]. In toddlers, nicotine and TSNA exposure from THS is up to 16 times higher [15] than adults through inhalation of SHS, and THS may cause hazardous health effects [4, 16–18].

Very little is known about the extent to which THS contributes to nonsmoking children’s overall levels of tobacco smoke exposure (OTS), defined as their combined THS and SHS exposure. Even less is known about the effect of OTS and THS on child health. Such knowledge is crucial so that remediation strategies can be developed to protect children from SHS and THS pollutants. Building on prior research and leveraging the experimental design from an active tobacco cessation trial of caregivers who smoke and their children (NIHR01HD083354), we will examine how home smoking behaviors contribute to THS and OTS pollution and exposure. Specifically, we will determine to what extent home THS contributes to OTS exposure over and above SHS and to what extent THS continues to linger and lead to OTS in homes with smoking bans and in homes after smokers quit. Targeted and novel nontargeted analyses of home dust samples will explore which types of THS pollutants are present. We will explore if child handwipe samples can provide valid markers of THS pollutants in the child’s home environment. Electronic medical record (EMR) review will be conducted to examine if THS and OTS levels are associated with child respiratory illness. Additionally, a repository of biological and environmental samples from smoke exposed children will be created for research on SHS, THS, and OTS.

### Aims and Hypotheses

#### Primary Aim 1

To examine the contribution of THS in children’s environments to their OTS exposure. Samples will be analyzed at baseline (T0) and 6-weeks after a smoking cessation intervention (T1) to assess THS pollution (nicotine from handwipe samples; dust NNK, nicotine, nicotelline) and biomarkers of child OTS (urinary cotinine, NNAL) and THS exposure (urinary NNAL/cotinine and N-Oxides/cotinine ratios).

##### Hypothesis 1.1

At T0, higher levels of THS pollution will be associated with higher levels of OTS and THS exposure markers, controlling for number of smokers and cigarettes smoked, and home smoking restrictions.

##### Hypothesis 1.2

At T1, children of quitters will have significantly lower levels of OTS exposure than at T0; T1 levels of THS exposure will be significantly above levels of children in nonsmoker’s homes.

#### Specific Aim 2

To determine the levels and composition of THS pollutants in house dust 6-weeks (T1) and 6-months (T2) after a cessation intervention. Dust will undergo analysis for tobacco carcinogens (TSNAs, PAHs), and novel nontargeted analyses to comprehensively characterize the mixture of chemicals that make up THS.

##### Hypothesis 2.1

Higher levels of nicotine and TSNAs in dust at T1 will be associated with continued smoking at T1, controlling for cigarettes smoked, home smoking restrictions, and T0 hand nicotine levels.

##### Hypothesis 2.2

Nontargeted analyses of dust from homes of those who quit or who have established complete smoking bans at T2 will have a higher proportion of novel secondary THS pollutants at T2 compared to T1. Targeted and nontargeted dust analyses from homes of continued smokers will have higher levels of THS pollutants and similar mixtures at T1 and T2.

##### Exploratory Hypothesis 2.3

Child hand nicotine levels may serve as tracers of other THS pollutants in the home and markers of THS exposure as determined by targeted and nontargeted analyses of dust and targeted analyses of urine samples at T1 and T2.

#### Specific Aim 3

To examine child and smoking factors associated with THS and OTS levels and to examine if levels are associated with respiratory-related diagnoses, interventions, tests, and Pediatric Emergency Department (PED)/Urgent Care (UC) visits.

##### Hypothesis 3.1

Higher levels of THS and OTS will be present in children who: are younger, live in apartments, live with more smokers and more cigarettes smoked; have higher rates of respiratory related diagnoses (e.g., asthma), interventions (e.g., oxygen, antibiotics), and positive tests (e.g., pneumonia).

##### Hypothesis 3.2

Children of smokers will have higher rates of pediatric emergency or urgent care visits than children of nonsmokers.

#### Secondary Aim 1

To create a repository of biological and environmental samples of smoke exposed children and their home environments.

## Methods/Design

### Overview of study design

The proposed study will leverage the study design, operational infrastructure, and collection and storage of samples of an ongoing, IRB approved, two-group randomized controlled trial (R01HD083354) [19]. This trial is designed to test the efficacy of a Screening, Brief Intervention, and Referral to Treatment (SBIRT) tobacco cessation intervention compared to an active control condition on caregivers who smoke who bring their child to the PED or UC at Cincinnati Children’s Hospital Medical Center (CCHMC). Caregivers randomized to the SBIRT condition receive face-to-face, tailored counseling that focuses on the child’s illness, the importance of reducing child SHS, caregiver smoking cessation, and the option to receive 12 weeks of nicotine replacement therapy. Caregivers randomized to the Healthy Habit Control (HHC) condition receive healthy lifestyle information that focuses on improving their child’s health by encouraging the “Let’s Go! 5-2-1-0” health practices [20]. Child participants are patients in the PED/UC who are nonsmokers and present with a potentially SHS-related complaint (e.g., cough, congestion). In this project, we will conduct secondary analyses on caregiver assessments and child biological and environmental samples that are not being analyzed from R01HD083354, and child clinical data abstracted from the EMRs of child participants.

Electronic self-report assessments are conducted in-person at baseline (T0) as part of R01HD083354 and via email or phone with verification of responses in-person during home visits at 6-weeks (T1) on all participants. For this project, home visits also occur at 6-months (T2) on 30 caregivers who are continued smokers and 30 caregivers who report that they have quit or have established complete smoking bans at T2. Consent includes permission to collect and store additional and leftover samples in a secure repository for potential future research. See Table 1 for the data collection and analysis plan by research aim.

**Table 1:** Schedule of enrollment, interventions, and assessments

Time Point	Study Period			
	Enrollment	Allocation	Post-allocation	
	Baseline T0	Baseline T0	6-weeks T1	6-months T2
Enrollment				
Eligibility Screen	X			
Informed Consent/Assent	X		X	
Allocation		X		
Interventions				
Intervention Group (SBIRT)		X		
Control Group (HHC)		X		
Assessments				
Sociodemographics	X	X		
Smoking history, Smoking Behavior, quit attempts		X	X	X
Parent reported Child TSE		X	X	X
EMR Review of Children TSE-related healthcare visits		X	X	X
Verification of quitting (exhaled carbon monoxide)			X	X
Verification of Child SHS exposure (urinary cotinine)		X	X	X
Sample Collection				
Urine		X	X	X
Handwipes		X	X	X
Dust			X	X
Air Monitor				X^a^

^a^Air Monitor is picked up approximately 1 week after T2

### Participant Inclusion/Exclusion Criteria

Adult participants are required to meet the following criteria in order to be eligible for enrollment in the trial:

1. Children and caregivers must have been enrolled at the T0 PED/UC visit as part of R01HD083354
2. Age 18 or older
3. Daily smoker at T0
4. English literate
5. Have a working cell or landline number
6. Caregivers and children must have a complete set of data and samples defined as: (a) caregiver assessments plus: (b) child handwipes and (c) child urine at T0 and at T1 and (d) house dust at T1

Exclusion criteria:

1. Tobacco chewers only
2. Live more than 50 miles away
3. Plan to move with no permanent address within 6 months of enrollment at T0
4. Child is an active smoker or marijuana user
5. Child is tracheostomy dependent

### Study Procedures

#### Setting

Study procedures occur at the PED/UC of CCHMC and at study participants’ homes.

#### Recruitment and study flow

Trained Clinical Research Coordinators (CRCs) obtain informed consent for participation in T0 and again at the T1 visit for participation in Aim 2. During the home visit at T1, the CRC will explain study procedures relevant to Aim 2 to eligible caregivers. Since we need 30 caregivers who are continued smokers and 30 who have quit smoking or established complete smoking bans, caregivers are told that they may be asked to participate depending on their answers to questions at T2. Informed consent will be obtained by the CRC; children over age 11 will provide assent. A secure database will be used during enrollment times, which will include all patients approached, with information on their age, sex, race, chief complaint, enrollment status, and reasons for non-enrollment (if applicable).

#### Randomization and treatment conditions

Participants were already randomized at T0 and remain in either the SBIRT or HHC condition for the entire study. Components of the SBIRT and HHC condition are reported elsewhere [19].

### Assessment procedures

#### Measures

Caregiver self-assessments are completed at T0, T1, and T2. Assessments include measures of sociodemographics; smoking history and smoking behavior: current tobacco use, electronic cigarette use, number of household smokers, numbers and location of cigarettes smoked; and house type (e.g., multiunit or detached home). We will extract CCHMC EMR data from T0 including: demographics; past medical history and respiratory-related clinical data such as ICD-10 diagnoses; clinical test and radiograph results (if any); interventions; respiratory assistance; medications; and disposition. For Aim 3, Hypothesis 3.2, we will also examine the frequency of PED or UC visits to CCHMC for the 200 children 6-months to 17 years old that are part of Aim 1 at T0 and for 6-months prior to T0, using CCHMC’s EMR system Epic. This PED and UC visit data will be compared to the same Epic data from an age-and 6-month time period-matched sample of 200 non-tobacco exposed children that were identified during R21CA184337 [21].

### Primary and secondary outcome variables

We will obtain the following primary outcomes from the sample analyses in Aim 1 and Aim 2: handwipe nicotine; dust nicotine, nicotelline, TSNAs; urinary cotinine, NNAL; urinary NNAL/cotinine and N-Oxides/cotinine ratios. The primary outcomes for Aim 2 are the same as Aim 1 and also include dust PAHs and the results of untargeted analyses of dust. We will obtain the following secondary outcomes in Aim 3: respiratory-related diagnoses, interventions, tests, and PED/UC visits.

### Covariates, mediators, and moderators

Covariates that will be considered in all analyses include home type (i.e., multiunit home/apartment, single-family home), child age and sex.

### Sample collection and chemical analysis by sample type

#### Sample collection, specimen handling, and storage

Standardized protocols are used to collect all data and samples at all visits. During the home visits, CRCs will obtain detailed information about house characteristics (e.g., measurements) and the room in which samples are collected. Home environmental samples (i.e., home dust, air monitors) will be collected in the room where the most smoking occurs (for homes with smokers) or in the room where the child spends the most time (for homes with nonsmokers or homes with smoking bans). Study staff and participants are blinded to results of tests. All samples will be placed on ice and then processed and stored at -80°C at CCHMC until ready for shipment. Samples will be shipped on dry ice via overnight shipping to the Environmental Health Analytic Chemistry Lab at San Diego State University (handwipes, dust, air monitors) and the Clinical Pharmacology Lab at the University of California San Francisco (urine for nicotine, TSNA, and nicotelline metabolites). Once at these laboratories, samples will be stored at -20°C until ready for analysis.

#### Repository collection

To facilitate future novel scientific discovery of this unique pediatric population of tobacco smoke exposed children, we will collect and store de-identified, unanalyzed environmental and biological samples at -80°C and corresponding datasets at T0-T2 on all participants who consented to this at T0.

#### Biological and environmental sample collection and chemical analyses

##### Urine samples: OTS

For Aims 1 and 2, approximately >7 ml of urine is collected and aliquoted into cryovials; samples will be analyzed for cotinine, NNAL and nicotelline N-Oxides, which are measures of OTS. Cotinine is a biomarker of recent TSE [22]. NNAL is a metabolite of the tobacco specific nitrosamine NNK, a marker of OTS (SHS and THS) in nonsmokers, and a biomarker of exposure to TSNA carcinogens [23]; the half-life is approximately 10 days. NNK in dust is a marker of THS. N-Oxides are metabolites of nicotelline. In dust, nicotelline is a THS marker for particulate matter (PM) derived from tobacco smoke [24]. We will derive biomarkers for THS exposure based on ratios of TSE biomarkers: NNAL/cotinine and N-Oxide/cotinine. Urine samples will be analyzed by LC-MS/MS; analytical methods are published [24–26].

##### Handwipes: THS pollution

Handwipes will be analyzed to measure nicotine, a marker of THS pollution on surfaces in the child’s home environment. THS handwipe procedures have been established by us and used with children and adults [5–7, 27, 28]. Taking a sample of THS surface nicotine involves: (1) preparing a solution of distilled water and 0.1% ascorbic acid (i.e., vitamin C); (2) wetting a screened cotton round with the solution; (3) wiping the palm and fingers of the dominant hand; and (4) storing the wipe in a vial for further analysis. Hand nicotine levels are reported in nanograms (ng) per wipe. Following Quintana, et.al [27], field blanks are collected to correct for any contamination from extraneous sources. Wipe samples will be stored at -20°C in the dark until analysis for nicotine. Nicotine in wipe samples will be analyzed by LC-MS/MS. The sample preparation and instrumental conditions have been reported previously [28–30]. We will measure the size of children’s hands and assess the last time their hands were washed.

##### Dust Samples: Targeted Analyses

Dust samples will be analyzed using targeted methods to quantify levels of nicotine, TSNAs (NNK, NNN, NAT, NAB), and nicotelline. Nicotine, TSNAs, and nicotelline are tobacco-specific compounds representing gas and particulate phases of tobacco smoke. When detected in dust, they serve as environmental tracers of THS that have accumulated in settled house dust. Dust samples will be collected using a standardized protocol. A 1m×1m area (or from a larger area, if needed, to collect approximately 1cm of dust in collection bottle) with a High-Volume-Small Surface-Sampler (HVS4, CS3 Inc., Venice, FL) into methanol-washed Teflon bottles. Dust samples will be weighed and sieved with a stainless steel, methanol-washed, 150 micrometer mesh sieve to remove large debris such as pet hairs, and weighed again. Sieved dust samples will be analyzed by isotope dilution LC-MS/MS. Detailed methods have been published elsewhere [28, 30].

##### Dust Samples: Nontargeted Analyses

We will determine the concentration of 16 priority PAHs as identified by the U.S. Environmental Protection Agency, including benzo[a]pyrene, chrysene, pyrene, fluorene, fluoranthene, and naphthalene [31, 32]. Additional toxic PAHs will be identified during the GCxGC/TOF-MS nontargeted analysis. We will employ a novel nontargeted analytical technique developed by Dr. Hoh as a core tool [33–35]. This method is based on a comprehensive two-dimensional gas chromatography coupled to time-of-flight mass spectrometry (GCxGC/TOF-MS). GCxGC/TOF-MS has a superior ability to identify compounds based on the enhanced sensitivity and chromatographic separation power provided by the GCxGC chromatography system and the simultaneous collection of ions at a fast data acquisition rate by the TOF-MS [36, 37].

##### Air Samples

Air samples will be placed at T2 in the room chosen (see *Sample Collection, Specimen Handling, and Storage,* above). A passive diffusion monitor badge will be used, consisting of a modified 37mm 3M Organic Vapor Monitor (3-M, St. Paul, MN) with a glass fiber filter coated with a glycerol/phosphoric acid mixture (filter collector will be modified from Kuusimaki, et al. [38]). The CRC will tape an active monitor to a wall five feet above ground, out of children’s reach and away from windows, corners, and doors. Inactive monitors will be placed in all other rooms to enhance reporting accuracy. CRCs will conduct home visits approximately 7 days later to retrieve the monitors, and the minutes the badge was placed will be recorded. Field blanks will be collected and 10% will be analyzed. Samples will be analyzed by isotope dilution LC-MS/MS; extraction will take place as for handwipes, see above [28, 30].

#### Participant Retention Strategies

Multiple strategies will be used to retain the sample including: 1) Adequate incentives for their time: participants will be paid with increasing compensation of $30 at T0, $50 at T1 ($20 for the questionnaire, $30 for the home visit), and $65 at T1 ($30 for the questionnaire, $35 for the home visit), plus a bonus $10 if they complete both of the follow-up assessments; 2) Conducting home visits on caregivers and children at T1 at T2 to assess outcomes and obtain samples, making study participation convenient for participants; 3) attempting to contact participants up to 10 times after T0 to arrange follow-up visits; 4) conducting “drop-in” unscheduled home visits on participants we are unable to contact; and 5) options to complete caregiver assessments via email.

#### Data quality control

Quality control procedures will be implemented to assure accurate and complete recording of survey responses, collection of environmental and biological samples in the field, the analysis of samples in the laboratory, and database entry. All data will be screened for missing and out-of-range responses, inconsistencies, and distributional properties. Depending on the statistical model used for a particular analysis, distributional assumptions will be examined. If necessary, response variables will be transformed for analysis (e.g., log transformation to address heterogeneous variances) and alternative statistical models will be evaluated. We will conduct sensitivity analyses comparing findings and conclusions from alternative approaches, including General Estimating Equations when examining changes over time, to optimize the number of subjects included regarding the dependent variable of interest. Validation samples will be investigated to determine possible contamination of samples in the field and sources of error in the lab. This is a critical part of quality assurance efforts when collecting environmental and biological markers of SHS/THS pollution and exposure in the field. Statistical analyses will be performed using the latest versions of STATA, R, SAS, and SPSS software packages [39, 40]. The Type I error rate will be set at α=.05 and corrections will be performed as necessary.

### Statistical analysis plan

#### Power analysis and sample size calculations

Sample sizes are based on our prior research of THS pollutants and exposure in homes of smokers, nonsmokers, smokers who quit, and nonsmokers who move into smoker’s homes. From this research, we expect: (a) standardized mean differences comparing dust and surface nicotine levels and urinary cotinine between homes with active smoking and nonsmokers to be d=1.5-2.0 and between homes of smokers with and without indoor smoking bans to be d=0.8-1.0; and (b) the proportion of variance in exposure accounts for 3-7% of the overall variance accounted for (R^2^=0.25 to 0.40) [41, 42]. Using GPower, we determined that under the conservative scenario (lower boundary estimates of above ranges), *N*=200 at T0 and T1 will yield power>0.80. For the more optimistic scenario (upper boundary estimates of above ranges), power will be >0.90.

#### Aims, hypotheses, statistical analysis

The data-analytic approach relies on linear models for cross-sectional and longitudinal designs. Each model will evaluate important potential moderating and confounding conditions, including the child age, sex, and home (e.g. type, size, flooring, number of rooms). We will examine how attrition may affect generalizability of findings and statistical models may require adjustment to control for potential biases.

##### Primary Aim 1

A multivariate multiple linear regression model (command mvreg in Stata) will be used to investigate if T0 hand nicotine levels (explanatory variable) are independently associated with exposure (three response variables: cotinine, NNAL, N-Oxides), controlling for child age, child sex, number of smokers and cigarettes smoked (including e-cigarettes), smoking bans, and type and size of home. Categorical explanatory variables will be dummy-coded and interaction effects with child sex and age will be explored. Overall model fit statistics and omnibus tests (R^2^, RMSE, omnibus F) will be evaluated, and specific hypothesis tests will be conducted for each of the explanatory variables of interest. A similar multivariate multiple linear regression model will also be estimated to determine if T0 hand nicotine levels are independently associated with THS exposure, controlling for child age, child sex, number of smokers and cigarettes smoked, smoking bans, and type of home.

To investigate if OTS levels in children of caregivers who have quit or in children of caregivers who have established smoking bans differ from known OTS levels of nonsmokers, we will analyze whether the observed mean OTS levels in the model above differ from the known levels of cotinine and NNK from published studies (controlling for child age and sex). To compare OTS levels at T1 and T0, we will use linear mixed models (command mixed in Stata) with OTS markers (cotinine, NNK, nicotelline) as response variables, subjects as the random factor, and time as a fixed factor (T0, T1).

##### Primary Aim 2

Based on T1 data only, we will examine how changes in smoking behavior affected THS pollution in dust. This hypothesis will be tested using a multivariate multiple linear regression model with TSNA levels in dust as the response variables and change in smoking behavior, number of remaining smokers, number of cigarettes smoked, and home smoking restrictions as explanatory variables. Categorical explanatory variables will be dummy-coded and possible interaction effects with child sex and age will be explored. Overall model fit statistics and tests will be evaluated and specific hypothesis tests will be conducted for each of the explanatory variables of interest. If standard ordinary least squares models are found to not be appropriate, we will rely on corresponding maximum likelihood estimation models and their fit indices and hypothesis tests.

At T2, we will collect data from *N*=30 homes of quitters or those who have confirmed home smoking bans (based on air nicotine levels) and *N*=30 homes of continued smokers. Because of the relatively small sample size, mean levels of groups of pollutant concentrations (e.g., oxy PAHs, nitro-PAHs, TSNAs) will be compared at T1 and T2 via paired sample parametric or nonparametric tests.

##### Primary aim 3

We will extract clinical data from the EMRs of the T0 visit of the 200 participants from Aim 1. We will use EMR data to assess frequencies and types of emergency care visits that these 200 smoke exposed children had for 6-months prior to T0 compared to Epic data from an age and 6-month time period-matched sample of 200 non-smoke exposed children (from R21CA184337) [21]. Descriptive statistics will be examined, including frequencies and percentages for categorical variables and means and standard deviations for continuous variables. Missing values and outliers will be checked and verified. The distributional properties of the outcome variables defining THS and OTS exposure will also be examined to see if transformation will be required for analysis purposes. Multiple linear regression models and t tests will be used to examine associations between home environment and THS and OTS levels, respectively, and EMR data from T0, including sociodemographics, past medical history, ICD-10 diagnosis, positive ancillary test results, interventions, medications given, and disposition. Multiple linear regression models will also be conducted to investigate factors predicting exposure levels. Linear models for dichotomous (logistic) and count outcomes (Poisson or Negative Binomial) will be used to examine the association between exposure and respiratory-related illness and health care utilization variables, controlling for child age, home type and relevant sociodemographic and clinical variables.

##### Missing data

We will follow guidelines as per Little et. al [43], to minimize attrition and optimize the use of all data, although there may still be bias with respect to the selected subjects. Collection of sociodemographic information on those included and excluded allows us to adjust statistical models for appropriate covariates.

### Data safety monitoring plan (DSMP)

We do not anticipate risk of interventions; however a DSMP has been maintained for all participants as part of R01HD083354. Validity and integrity of the data is being ensured by appropriate research design, use of pretested tools for data collection and by quality assurance.

### Data confidentiality and archiving

We will strictly maintain the privacy, anonymity, and confidentiality of the data and samples we have and are collecting. In order to protect the safety of participants, study data and samples are protected by a Certificate of Confidentiality and are stored securely at CCHMC. Coded identification numbers are used to anonymize and depersonalize the data. The linking code, electronic data files and any paper forms are stored in a separate location under password protections or lock and key. Access to the data and samples are limited to authorized study staff.

### Dissemination policy

The project results will be published regardless of the outcome upon approval from all investigators.

### Project update

Baseline data collection and implementation of interventions commenced April 2016 and are currently ongoing. Initial follow-up home visits commenced in May 2016 and are currently ongoing.

## Discussion

This study is based on a growing body of research demonstrating that exposure to tobacco smoke toxicants among nonsmokers is not limited to inhaling SHS, the mixture of exhaled mainstream smoke and sidestream smoke. Nonsmokers may also be exposed to the residue of SHS compounds left behind on surfaces, in dust, and embedded in materials long after cigarettes have been smoked. Because of the physical and chemical properties of its constituents, SHS easily spreads throughout different rooms of a home, to neighboring apartments, and to different floors of a larger building. Commonly referred to as THS, this residue is pervasive throughout indoor environments where tobacco products have been used and can be found in carpets, pillows, walls, desks, upholstery and even on the hands of nonsmokers living in such THS polluted environments. The pervasiveness of THS in dust and on surfaces allows for exposure through dermal transfer and ingestion, pathways typically not considered for SHS exposure. Because young children are often in closer contact with their physical environment than adults (e.g., crawling playing on the ground, pica behavior), they are at particular risk of exposure to THS that accumulates in dust and on surfaces.

Utilizing this unique sample of smoke-exposed children, this project will be the first to differentiate the individual and collective contribution of SHS and THS to overall OTS. The results will identify the types, levels, markers of, and health risks of tobacco toxicants. This research can potentially change how we protect children from all forms of tobacco smoke exposure, by recognizing that SHS and THS needs to be addressed separately as sources of pollution and exposure. Results will be used to develop remediation strategies to more completely protect children from dangerous tobacco toxicant-related exposures. The effect of the implementation of these THS reduction strategies on OTS levels and child health will be tested in future trials.

## Abbreviations

CCHMC: Cincinnati Children’s Hospital Medical Center
CRC: Clinical research coordinator
DSMP: Data safety monitoring plan
EMR: Electronic medical record
GCxGC/TOF-MS: Comprehensive two-dimensional gas chromatography coupled to time-of-flight mass spectrometry
HHC: Healthy Habit Control
IRB: Institutional Review Board
LC-MS/MS: Liquid chromatography-tandem mass spectrometry
NAB: 1-nitrosoanabasine
NAT: N'-nitrosoanabasine
Ng: Nanograms
NNAL: 4-(methylnitrosamino)-1-(3-pyridyl)-1-butanol
NNK: Nicotine-derived nitrosamine ketone
NNN: N'-nitrosonornicotine
OTS: Overall tobacco smoke
PAH: Polycyclic aromatic hydrocarbons
PED: Pediatric emergency department
PM: Particulate Matter
SBIRT: Screening, Brief Intervention, and Referral to Treatment
SHS: Secondhand smoke
THS: Thirdhand smoke
TSNA: Tobacco-specific nitrosamines
UC: Urgent care
